# Hidden Markov models reveal temporal patterns and sex differences in killer whale behavior

**DOI:** 10.1038/s41598-019-50942-2

**Published:** 2019-10-18

**Authors:** Jennifer B. Tennessen, Marla M. Holt, Eric J. Ward, M. Bradley Hanson, Candice K. Emmons, Deborah A. Giles, Jeffrey T. Hogan

**Affiliations:** 1 Conservation Biology Division, Northwest Fisheries Science Center, National Marine Fisheries Service, National Oceanic and Atmospheric Administration, Seattle, WA USA; 2 Lynker Technologies, Leesburg, VA USA; 3 Department of Wildlife, Fish, & Conservation Biology, University of California, Davis, CA USA; 4 Cascadia Research Collective, Olympia, WA USA; 5 Present Address: University of Washington, Friday Harbor Laboratories, Friday Harbor, WA USA

**Keywords:** Behavioural ecology, Conservation biology, Evolutionary ecology

## Abstract

Behavioral data can be important for effective management of endangered marine predators, but can be challenging to obtain. We utilized suction cup-attached biologging tags equipped with stereo hydrophones, triaxial accelerometers, triaxial magnetometers, pressure and temperature sensors, to characterize the subsurface behavior of an endangered population of killer whales (*Orcinus orca)*. Tags recorded depth, acoustic and movement behavior on fish-eating killer whales in the Salish Sea between 2010–2014. We tested the hypotheses that (a) distinct behavioral states can be characterized by integrating movement and acoustic variables, (b) subsurface foraging occurs in bouts, with distinct periods of searching and capture temporally separated from travel, and (c) the probabilities of transitioning between behavioral states differ by sex. Using Hidden Markov modeling of two acoustic and four movement variables, we identified five temporally distinct behavioral states. Persistence in the same state on a subsequent dive had the greatest likelihood, with the exception of deep prey pursuit, indicating that behavior was clustered in time. Additionally, females spent more time at the surface than males, and engaged in less foraging behavior. These results reveal significant complexity and sex differences in subsurface foraging behavior, and underscore the importance of incorporating behavior into the design of conservation strategies.

## Introduction

Studying the behavior of organisms can yield insights that transcend disciplines, and is an urgent priority as global change imposes novel pressures on ecological patterns and processes^1–5^. For threatened and endangered populations, characterizing behavior can be integral to conservation outcomes^3^, yet particularly challenging for these populations. The rarity of such populations can make them paradoxically difficult to study^6^. Furthermore, behavioral studies are vulnerable to observer bias and the tendency to quantify behaviors through subjective lenses^7^. Additionally, for species that spend the majority of their time in habitats inaccessible to researchers, quantifying behavior can be especially challenging and confounded by time (i.e., only the behaviors that are observed are quantified)^5^.

Many populations of cetaceans (whales, dolphins and porpoises) are endangered globally^8^. Until recently it was difficult to obtain continuous observations of subsurface behavior to inform conservation efforts because these species spend the majority of their lives underwater. For endangered cetaceans that forage in groups, individuals need to balance the energetic gains of group-facilitated prey consumption with the metabolic costs of prey pursuit at depth^9–12^. The ways in which these species temporally structure their foraging activities to account for this paradox, however, are poorly understood yet fundamental to conservation.

We studied the foraging behavior of an endangered population of killer whales (*Orcinus orca*), which lives in the Northeastern Pacific Ocean, and spends the majority of its time in the inland and coastal waters along the west coast of North America between central California and southeastern Alaska. Individuals in the Southern Resident killer whale (SRKW) population spend their lifetimes in stable, matrilineal groups, and consume salmon, primarily Chinook (*Oncorhynchus tshawytscha*)^13,14^, that are routinely shared with group members^15^. Chinook salmon, the largest salmon species, provide a significant energetic return, but given that they occur deeper than other salmon species, up to several hundred meters^16,17^, SRKW must invest significant energy in the forms of breath holding and locomotion costs associated with fluking and drag^11,12^, in order to pursue these larger caloric payoffs. The challenge of acquiring sufficient Chinook salmon to meet caloric requirements is compounded by the fact that many Chinook salmon populations originating from the west coast of the United States and British Columbia are threatened or endangered^18^.

To study foraging behavior, past studies primarily relied upon observations of kills at the surface, collection of samples of prey floating in the water after kills, or collection of fecal samples indicating recent consumption e.g.^13,14^. Surface observations revealed distinct episodes of foraging activities, including increased swim speeds indicative of prey pursuit^14^, and aggregation behavior following prey capture, presumably to share prey among group members, particularly between dependent offspring and mothers. A recent study utilized movement variables of subsurface behavior to identify prey capture events, in order to explore sex differences in foraging ecology of fish-eating killer whales, revealing males made more prey capture dives than females, presumably to support the energetic requirements of a larger body size^19^. Currently, however, little is known about the extent to which these prey pursuits represent the culmination of continuous search efforts, or whether foraging occurs in distinct periods in space and time, allowing for other activities including recovery following metabolically costly deep dives, socialization, traveling, or other non-foraging activities. Additionally, given the sexual dimorphism between males and females and differences in metabolic rates, energetic requirements^12^ and foraging rates^19^, it is possible that males and females may partition behavioral activities or timing to minimize energy use while maximizing gain and meeting other needs including care of dependent offspring. Indeed, in many species of sexually dimorphic marine mammals, foraging behavior is sexually segregated^20–22^. Addressing these data gaps can inform management of the SRKW population in critcal habitat including the inland waters of Washington State and British Columbia, where many commercial and recreational interests coincide with the whales’ foraging activities from May through September.

We utilized multisensor biologging tags to characterize the subsurface behavior of SRKW. Specifically, tags were attached by suction cup to killer whales and recorded depth, movement, and sound, providing the opportunity to collect a continuous stream of data as the subjects traveled below the surface. To minimize the effect of subjective observer bias on behavioral categorization, we used hidden Markov models to systematically characterize latent behavioral states based on four movement and two acoustic variables computed from data recorded on the tags. This approach enabled quantification of behavioral states based on similarities identified through a latent Markov process rather than relying on subjective assignment by researchers. We then used these behavioral state allocations to test the hypotheses that (a) SRKW engage in distinct behavioral activities characterized by differences in their subsurface movement and acoustic behavior, (b) SRKW foraging behavior occurs in bouts, with distinct periods of searching and capture followed by rest, and (c) the probabilities of transitioning between behavioral states differ by sex.

Knowledge of the behavior of a species, the temporal patterning of activity budgets, and whether behavioral patterns differ by sex is valuable for guiding effective conservation^5,23^. The SRKW population was listed as Endangered in the United States^24^ and as a Species at Risk in Canada^25^. Though this population has been small (<100 animals) since 1975, recent declines have reduced the population to a near record low of 73 animals at present, prompting increased concern over its future viability^26,27^. One of the main threats to population persistence is availability and accessibility of prey^14,27,28^. Therefore, a better understanding of the temporal patterns of behavior, and whether foraging-related activities including shallow searching and deep prey pursuit differ by sex, will enable the design of effective SRKW conservation measures that promote foraging opportunities.

## Results

### Summary of deployments

We analyzed a total of 41.8 h from all 13 deployments (2010 = 9.3 h, n = 3; 2012 = 15.3 h, n = 6; 2014 = 17.2 h, n = 4) (Table 1). Mean analyzed time per deployment was 3.2 h (range = 0.6–6.9 h). We analyzed 3728 dives from these deployments. Females (f) and males (m) made a total of 1324 and 2404 dives, respectively (2010: 253 (f), 517 (m); 2012: 429 (f), 912 (m); 2014: 642 (f), 975 (m)). The number of dives analyzed per deployment ranged from 37 to 583 (Table 1).

**Table 1:** Summary of analyzed Dtag deployments on Southern Resident killer whales.

Date	Time on (hh:mm:ss)	Whale ID	Sex	Age (y)	Dur (h)	No. dives analyzed	Number of dives per state				
							1	2	3	4	5
2010-09-18	15:32:45	L72	F	24	0.62	37	0	16	15	0	6
2010-09-21	12:37:09	L83	F	20	2.53	216	0	86	27	0	103
2010-09-22	12:15:42	K33	M	9	6.18	517	34	183	97	107	96
2012-09-07	11:22:21	K33	M	11	1.57	153	11	42	36	64	0
2012-09-10	10:46:44	L95	M	16	6.94	583	10	95	111	307	60
2012-09-17	10:11:55	L84	M	22	2.11	176	2	1	60	42	71
2012-09-22	10:39:21	L91	F	17	2.56	214	12	58	46	78	20
2012-09-22	13:45:09	L47	F	38	0.51	56	1	15	14	26	0
2012-09-23	14:56:07	J28	F	19	1.63	159	0	76	35	48	0
2014-09-06	09:55:10	L113	F	5	5.61	583	11	159	100	55	258
2014-09-20	11:57:15	L85	M	23	6.48	510	15	45	116	195	139
2014-09-21	11:31:46	L91	F	19	0.68	59	4	18	25	10	2
2014-09-23	10:53:41	K35	M	12	4.47	465	20	126	91	217	11

### State classifications of diving behavior

**Figure 1:**
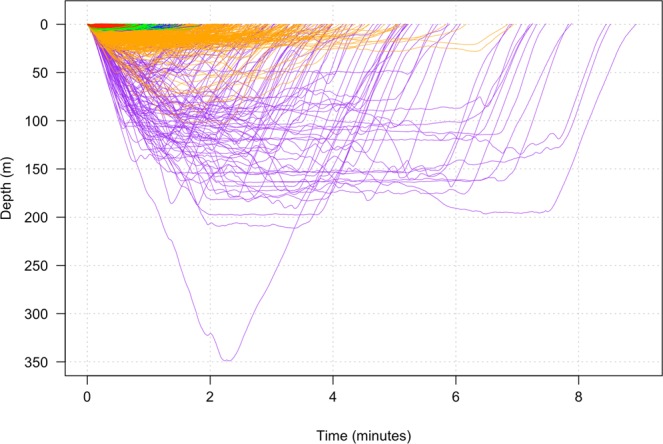
Hidden Markov modeling revealed five distinct behavioral states across all individuals. Dives (n = 3728) are plotted for all deployments (state 1 = purple, state 2 = red, state 3 = orange, state 4 = green, state 5 = blue).

**Figure 2:**
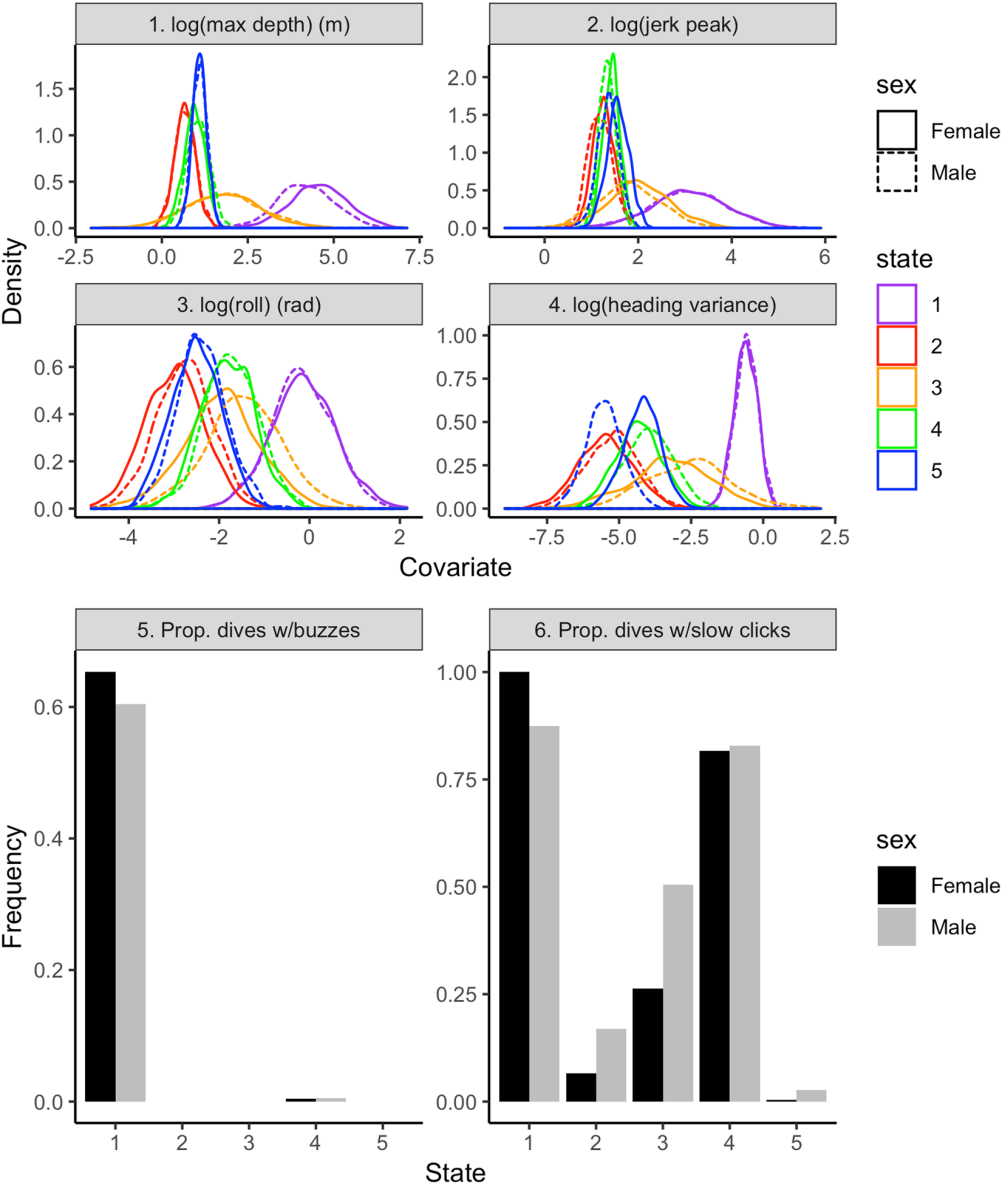
Fitted state-dependent distributions of the best model by sex.

**Figure 3:**
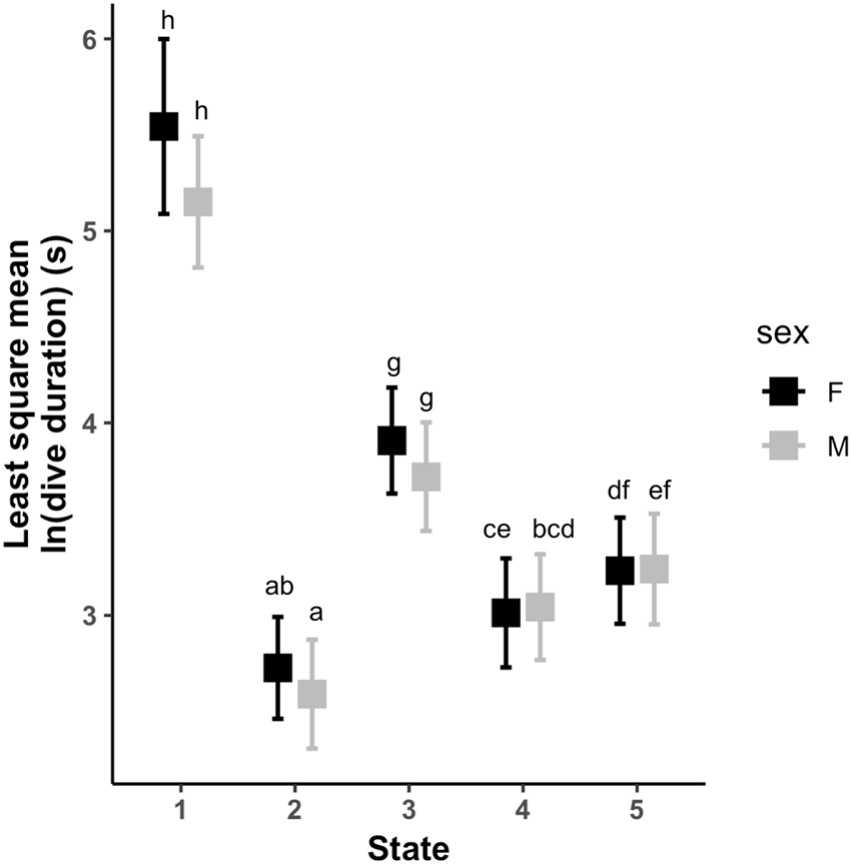
Differences in dive duration by state and sex. Boxes indicate least square mean values for females (black) and males (gray), whiskers indicate confidence. Significance, determined by a Tukey HSD test across all states and sexes, is indicated by unshared letters above bars.

The unit of analysis was a dive, defined as any departure from and return to the surface. The best model identified five underlying behavioral states in Southern Resident killer whales (Fig. 1, Supplementary Figs S1 and S2). These states were characterized by: (1) deep dives with common occurrence of buzzing, nearly ubiquitous occurrence of clicking, and large values of jerk peak, roll and heading variance, (2) shallow dives with no buzzing, uncommon clicking, and small values of jerk peak, roll and heading variance, (3) shallow to intermediate dives with no buzzing, some clicking, and small-to-moderate values of jerk peak, roll and heading variance, (4) shallow dives with virtually no buzzing, abundant clicking, small values of jerk peak and heading variance, and small-to-moderate values of roll, and (5) shallow dives, no buzzing, rare clicking, and small values of jerk peak, roll and heading variance (Table 2, Fig. 2). There was a significant effect of state on dive duration (linear mixed model: *F*_4, 3718_ = 761.2, *p* < 0.0001) (Fig. 3).

**Table 2:** Median, first and third quartile values of kinematic response variables and proportion of dives containing buzzes and clicks, by state and sex.

Sex	State	Response variable					
		Max depth (m)	Jerk peak	Roll (deg)	Heading variance	Buzz prop.	Slow click prop.
F	1	107.34 (86.35–135.56)	23.44 (12.91–36.07)	62.45 (26.13–85.65)	0.59 (0.39–0.75)	0.64	1
	2	2.06 (1.69–2.43)	3.48 (2.89–4.26)	2.86 (1.84–4.42)	<0.01 (0.002–0.008)	0	0.05
	3	6.49 (2.57–16.98)	7.33 (4.47–12.02)	8.02 (4.70–15.44)	0.04 (0.01–0.12)	0	0.27
	4	2.51 (2.14–3.03)	4.07 (3.67–4.82)	9.17 (5.75–13.16)	0.01 (0.01–0.02)	<0.01	0.82
	5	3.00 (2.63–3.46)	4.68 (4.02–5.56)	4.58 (3.13–6.50)	0.02 (0.01–0.02)	0	<0.01
M	1	91.78 (40.82–132.80)	23.27 (11.16–45.55)	52.71 (29.57–87.53)	0.61 (0.47–0.72)	0.62	0.89
	2	2.01 (1.68–2.38)	3.16 (2.80–3.73)	3.44 (2.25–5.46)	<0.01 (0.003–0.009)	0	0.17
	3	6.18 (3.02–16.38)	5.33 (4.14–8.08)	13.75 (7.71–24.61)	0.08 (0.03–0.22)	0	0.48
	4	2.96 (2.36–3.51)	3.78 (3.33–4.26)	10.31 (6.71–15.48)	0.02 (0.01–0.03)	<0.01	0.82
	5	2.94 (2.56–3.37)	3.88 (3.42–4.45)	5.73 (3.83–7.21)	<0.01 (0.002–0.006)	0	0.03

Within each cell of the four kinematic response variables, median is displayed on top and first and third quartile values are displayed on the bottom, from left to right, respectively.

### Transition probabilities

**Figure 4:**
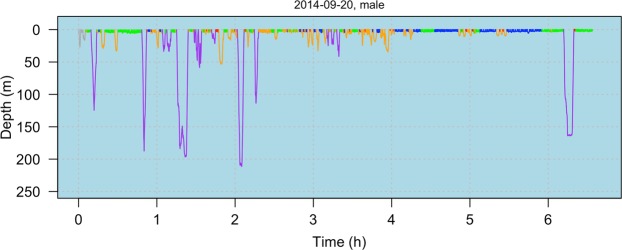
Dive profile of a deployment on a male killer whale, indicating behavioral state allocation over time. State 1 = purple, state 2 = red, state 3 = orange, state 4 = green, state 5 = blue. Gray dives indicate the omitted 5-min interval at the start of each deployment.

**Figure 5:**
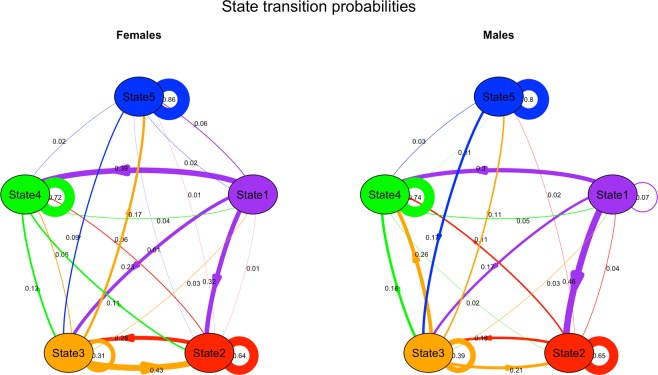
Transition probabilities between states for females and males. Line color indicates the state of origin, and line thickness scales positively with probability, which is indicated adjacent to the corresponding line.

For both females and males, the largest transition probabilities observed were for state persistence on the subsequent dive, indicating that behavior was temporally clustered, as expected (Fig. 4, 5, Supplementary Fig. S1). Females and males were most likely to persist in states 2, 4 and 5 (different types of shallow behavior). Persistence in state 1 (deep dives) never occurred for females, and was rare for males. For state 3 (shallow-intermediate dives), persistence was the most likely outcome for males but not females (Fig. 5).

For females and males, switching to state 1 was rare, whereas switching to state 2 was common from states 1 and 3. For females, switching to state 3 was moderately likely from states 1 and 2, less common from state 4, and rare from state 5. In contrast, after persistence in state 3, males were equally likely to switch to state 3 from any other state. Males were more likely than females to switch to state 4 from any state except state 1, suggesting males often returned to state 4 throughout a deployment. For both males and females, transitions into state 5 from states 1, 2 or 4 were rare, and infrequent from state 3 (Fig. 5).

### State allocations

**Figure 6:**
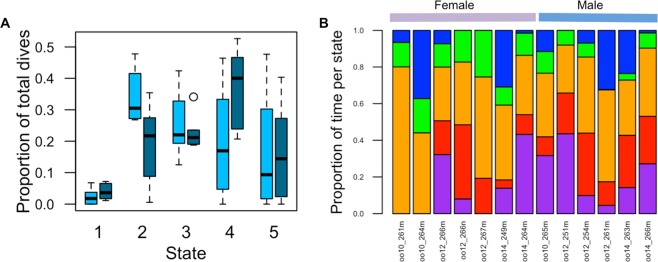
Summaries of behavioral state allocation for all deployments. (**A**) Boxplots of proportional occurrence of dives by state, per deployment, for females (light blue) and males (dark blue). Horizontal bars indicate medians, box edges are interquartile ranges, whiskers indicate minimum and maximum values, and outliers are plotted. (**B**) Proportion of time spent per state, per deployment, arranged by sex. State 1 = purple, state 2 = red, state 3 = orange, state 4 = green, state 5 = blue.

The multinomial logistic model supported the existence of differences between females and males in the frequency of different behavioral states (Table 3, Supplementary Table S1, Fig. 6A). The positive log odds ratio (with state 1 as the reference baseline) indicated both sexes were more likely to occur in states 2–4 than in state 1. Furthermore, the log odds ratio differed between sexes for all states except state 5, supporting the hypothesis of sex differences in state allocation, and was most different between sexes for state 2, indicating females were substantially more likely to occur in state 2 (respiration) vs. state 1 (deep prey pursuit) than were males.

**Table 3:** Number of dives, cumulative time in each state, and percentage of total time sampled by sex.

	State 1	State 2	State 3	State 4	State 5
Female	28 6641 (13.6)	428 6939 (14.2)	262 20579 (42.1)	217 4456 (9.1)	389 10235 (21.0)
Male	92 19311 (20.1)	492 6931 (7.2)	511 34928 (36.4)	932 22797 (23.8)	377 11999 (12.5)

Within each sex-by-state cell, number of dives is displayed on the top and cumulative time (seconds) on the bottom, followed by percentage of total time sampled in parentheses.

We found a significant difference between males and females in the proportion of time per state (χ^2^ = 7883.1, df = 4, p < 0.0001; Table 3; Fig. 6B). Females spent substantially less time than males in state 1 or 4, and notably more time than males in states 2, 3, and 5.

## Discussion

We used a latent Markov process to identify five temporally distinct behavioral states, demonstrating complexity in subsurface behavior of Southern Resident killer whales. From the most likely state sequence, we computed the probability of state persistence and state switching, revealing that behavior occurs in bouts. We show that females and males differ in their transition probabilities between states, and in the frequency and cumulative time in these states.

State 1 was characterized by deep dives, common buzz and nearly ubiquitous click occurrence, and large values of jerk peak, roll and heading variance. Additionally, state 1 dives had the greatest duration. Consistent with previous results^19^, state 1 is likely associated with close pursuit and capture attempts of salmonid prey, whereby prey are chased at depths typically greater than 30 m, producing substantial jerk movement due to abrupt acceleration and deceleration, as well as changes in musculature in the head region at prey interception^29–33^. Additionally, state 1 is associated with large values of roll, and large heading variance due to repeated direction changes^34^ while closing in on prey. Similarly, prey pursuit dives by Northern Resident killer whales, a partially-sympatric population with a similar diet^13^, tend to be deeper, longer, include a large roll angle, are more tortuous, and include faster swim speeds than non-pursuit dives^35^.

Dives allocated to state 2 were characterized by shallow depth, no buzzing and uncommon clicking, and small values of jerk peak, roll and heading variance. These dives were shortest in duration, reflecting their surface association, and typically followed intermediate to deep dives. These dives are likely associated with surface respiration, with small values of heading variance. The high probability of switching from state 1 to state 2, particularly in males, and moderate probability of switching from state 3 to state 2, as well as the high probability of persisting in state 2, supports the idea that state 2 dives likely function as respiration dives as in^35^ following depletion of oxygen stores during deep dives. The presence of occasional clicking by females and slightly more common clicking by males (Table 2, Fig. 2) suggests that a small amount of acoustic searching may be occurring during state 2 dives as well. State 5 dives were similarly characterized by shallow depth, no buzzes, rare occurrence of clicks, and small values of movement variables including heading variance, which is indicative of directional travel. These dives were short in duration but significantly longer than state 2 dives (Fig. 3), with the highest likelihood of persistence for any of the states. The notable occurrence of these dives in long bouts suggests that state 5 likely represents traveling.

State 4 was characterized by dives similar in depth and kinematics to states 2 and 5, but notably different was the nearly ubiquitous occurrence of clicks. Dives in this state were short as well, similar to those in states 2 and 5. State 4 is likely a prey searching state primarily, consistent with the acoustic behavior that precedes prey capture in many other toothed whales whereby an individual produces echolocation clicks that scan an area for possible targets prior to initiating a capture attempt e.g.^29,30,36–40^. Additionally, the tendency for these dives to occur in bouts further supports the interpretation that this is primarily a prey searching state. The absence of buzzes from nearly all dives in this state indicates these dives are unlikely to include capture attempts. It is important to note, however, that prey searching does not preclude the occurrence of other surface-associated behaviors that may occur simultaneously, such as traveling or socializing.

Finally, state 3 was characterized by intermediate depth, with no buzzes, relatively common click occurrence, and low to moderate values of jerk peak, roll and heading variance. These dives were intermediate in duration, suggesting there could be a variety of behaviors associated with dives in this state. The absence of buzzes indicates these dives are unlikely prey capture attempts, but it is possible that prey pursuit was initiated and then aborted at intermediate depth. This interpretation is unlikely to explain all dives classified to state 3, however, because approximately two thirds of female dives and half of male dives in this state did not contain clicks. It is possible that state 3 behaviors include socializing, such as engaging in tactile behavior and/or prey sharing, which could explain moderate values of heading variance, roll and jerk peak as individuals move vertically and horizontally and make physical contact with other group members^41^. Additionally, these behaviors may be best accomplished several meters below the surface, where positional alignment within a pod may be able to take on a three-dimensional formation whereas surface swimming arrangements are confined to essentially the same horizontal plane. Furthermore, acoustic interference with the surface can create zones that impede detection of signals^42,43^. Therefore, when communicating with individuals out of sight, using stereotyped pulsed calls and whistles with the majority of energy in mid-frequencies (1–20 kHz)^44,45^, it is possible that swimming approximately 15–25 m below the surface, as observed in many dives classified to state 3, may facilitate social behavior by optimizing passive listening for and active sending of important information.

For both females and males, behavioral bouts (sequences of consecutive dives of the same state) were common for states 2, 4 and 5, moderately common for state 3, and rare for state 1. This indicates that certain behaviors, particularly acoustic searching (state 4) and respiration and travel (states 2 and 5, respectively), are clustered in time. Surface travel behaviors are common in other social odontocetes e.g.^35,46,47^, supporting our finding of temporal patterning (bouts) of behavior. Indeed, subsurface behavior does not appear to be randomly distributed in time, but rather is temporally clumped, likely driven by other factors, including prey distribution^48,49^, bathymetry features that may aggregate prey^50,51^, time of day^51,52^, oceanographic parameters^53^, aerobic dive limits^54^ and anthropogenic pressures^55–58^. It is not possible to tease apart the relative importance of each of these factors in this study. However, the extreme sociality of resident-type killer whales likely plays a considerable role in mediating behavioral patterns. Resident-type killer whale movements are coordinated at a coarse scale, with pods often traveling together, or breaking apart briefly to reunite a few hours or a few days later. Furthermore, because of the existence of kin-directed prey sharing among resident-type killer whales^15^, coordinating behavior to maximize foraging success would benefit the population^59^. Indeed, studies have revealed synchronicity in diving behavior in other cetaceans^41,60–63^.

Notably, persistence in state 1 (final pursuit and capture attempt) between consecutive dives was rare. There are at least two, non-mutually exclusive explanations for this finding. First, deep diving for prey pursuit incurs significant energy expenditure through (1) the depletion of oxygen stores during breath-holding, (2) the kinematics of chasing prey including rapid acceleration and deceleration and sudden direction changes, and (3) withstanding the physiological effects of extreme pressure at depth. Rather than completing costly consecutive dives, individuals may enhance their overall foraging effectiveness by recovering near the surface (demonstrating behavior such as in dives classified to state 2) before initiating another prey pursuit dive. Indeed, many deep-foraging cetaceans make several recovery dives at the surface before returning to depth e.g.^35,46,64,65^. The second explanation for the paucity of state 1 persistence may be that a prey item is often captured during a deep foraging dive^19^. Upon capturing a prey item, it is then typically shared among pod-members near the surface^15^, precluding the occurrence of consecutive deep dives. If prey are not captured during pursuit, it is possible that their heightened vigilance may make consecutive deep prey pursuit dives less likely to be executed due to their potentially reduced likelihood of success.

We found sex differences in state switching behavior. Males were more likely than females to switch to acoustic searching (state 4) from any state except deep prey pursuit (state 1), suggesting males often returned to acoustic searching behavior. This may be driven by key deployments in which females did not engage in any deep prey pursuit behavior. Additionally, females were more likely than males to engage in non-foraging behavior, and spent notably more time in surface respiration, intermediate depth or traveling states than males, who spent substantially more time searching for and pursuing prey. It is possible that female foraging behavior is temporally compartmentalized, due to the time demands of caring for calves or other offspring or kin e.g.^66,67^, and due to the potentially increased costs of transport for females with calves^4^. Alternatively, since adult males are larger than females or juvenile males, and have the largest metabolic requirements in the population^12^, they may need to forage more often than females or juvenile males^19^ to meet their metabolic requirements, and consequently may engage in searching behavior more often. It is important to note our conclusions are limited by the sample size of deployments greater than three hours on females. We cannot rule out the possibility that collecting a greater number of longer deployments on females could reveal more equal time budgets of state allocation. Nonetheless, our results are consistent with known sex differences in prey capture and prey pursuit in Southern Resident killer whales^19^.

This investigation utilized Hidden Markov models to characterize the subsurface behavior of a population of killer whales, to shed light on the ways in which group foraging cetaceans temporally structure their foraging activities and to determine whether females and males differ in their behavioral patterns. We revealed that killer whale subsurface activity budgets are complex, involving at least five distinct behavioral states, and that females and males differ in subsurface activity, with females generally engaging in less foraging behavior than males, though we cannot rule out the possible influence of small sample size on this sex difference. We demonstrated that foraging is more than intermittent deep dives to acquire prey, following the general sequence: (1) an event causes a transition from the intermediate depth or traveling states into active searching at the surface using echolocation, (2) the individual identifies a potential target, and initiates a deep dive to investigate further or engage in initial prey pursuit, (3) the individual either abandons the investigation or pursues the target and initiates a chase and potentially a successful capture, and (4) the individual returns to the surface and continues searching or transitions to respiration, traveling or socializing behavior. Consequently, these findings underscore two important points. First, when determining how environmental or human factors may impact foraging, it is important to consider disturbance to multiple foraging-related behaviors, not just to the ultimate prey capture. Second, it is not possible to completely assess whether animals are engaged in foraging activities from surface observations alone. Studies that categorize behavior through focal observations may not be able to resolve differences between dive types, so they may not be able to quantify disruptions to foraging, for example impacts on searching effort, an integral component of foraging. Indeed, we demonstrate that searching appears kinematically similar to respiration or traveling, and is distinguished primarily by the presence of echolocation. Thus, studies quantifying human impact on cetacean foraging should combine surface observations with quantification of subsurface behavior, including both kinematic and acoustic data when possible. This approach, combined with modeling hidden states using latent Markov chains, can provide a valuable method for identifying patterns in behavioral data that can inform management of threatened and endangered species.

## Methods

### Data collection

We deployed multi-sensor biologging tags (‘Dtags’)^68^ on SRKW in the Salish Sea during September 2010 and 2014 (Version 2) and September 2012 (Version 3). Dtags were equipped with stereo hydrophones, triaxial accelerometers, triaxial magnetometers, and pressure and temperature sensors, and recorded subsurface acoustic and movement behavior. Dtags sampled audio at 192 kHz and depth, movement and temperature at 50 Hz (Version 2), or sampled audio at 240 kHz and depth, movement and temperature at 200 Hz (downsampled to 50 Hz during post-processing; Version 3). Dtags stored data to onboard flash memory, contained a VHF beacon to enable tag recovery, and were programmed to release before local sunset, if they had not already fallen off naturally. Tagging was conducted under federal research permits in the U.S.A. (NMFS No. 781–1824/16163) and in Canada (DFO SARA/Marine Mammal License No. MML 2010-01/ SARA-106B), and approved by the Northwest Fisheries Science Center’s Institutional Animal Care and Use Committee.

Twenty-three Dtags were attached by suction cup to the dorsal side of 21 killer whales using a 7 m carbon fiber pole held by a tagger standing on the bow pulpit of a 6.7 m rigid-hulled inflatable research vessel. Animal reactions to tagging consisted primarily of mild to moderate behavioral changes, and included flinching or diving for up to a few minutes, but resumed previous behavior within five minutes of tagging. Animals were tagged opportunistically, while ensuring a balanced representation between sexes. All animals were >1 year old. No animals were tagged multiple times within the same year, but two animals were tagged twice in different years. Focal follows of the tagged animal were conducted for the duration that the tag remained on the animal, described elsewhere^19,69^. These focal follows allowed for identification of changes in tag orientation for use in data calibration, and to facilitate tag retrieval. All tags were attached during only daylight hours.

### Data processing

Following tag retrieval, data were downloaded and custom software (the 2014 Dtag Toolbox, www.soundtags.org/dtags/dtag-toolbox) along with custom scripts in Matlab version 2016a (The Mathworks, Natick, MA, USA) were used to calibrate acoustic and movement sensor data and convert pressure data to temperature-corrected depth, following established methods^68^, see details in^19,69^. All acoustic audits of sound files were conducted by the same experienced researcher. We excluded nine deployments due to acoustic quality. For these deployments, excessive noise from water flowing past the hydrophones, often due to suboptimal tag placement, prevented acoustic variables from being computed. Additionally, we excluded the first deployment due to inconsistencies in data collection methods and hydrophone gain settings with the rest of the deployments. We down-sampled pressure data to 5 Hz, and applied a custom dive detector that identified a dive as the interval containing a departure from the surface (0.5 m), a maximum depth ≥1 m, and a return to the surface. Due to the relatively high sample rate in the pressure data, occasionally noise in the pressure data caused false detections. Therefore, we checked all dive results manually, and corrected dives as needed. We excluded dives that began within the first five minutes of tag attachment in order to account for any short-term behavioral responses to tagging. We confirmed that this was a conservative estimate of the duration of behavioral responses to tagging, by visually inspecting all dive profiles.

Dive was the unit of analysis in this study, defined as the time between successive respirations at the surface. For each dive, we calculated start and end times of the whole dive and of the bottom phase (≥70% of maximum depth), following methods in Arranz *et al*.^33^. Next, we computed six variables for each dive: *Maximum depth*, the deepest point of a dive (m); *Jerk peak*, the maximum peak in the jerk (rate of change of acceleration); *Roll*, the median of the absolute value of the animal’s roll (dorsal-ventral); *Heading variance*, the circular variance in the animal’s heading (left-right); *Buzz presence*, the binary presence of echolocation pulses with an inter-click interval ≤10 ms within a click bout; *Slow click presence*, the binary presence of echolocation clicks with an inter-click interval >100 ms within a click bout. Both acoustic variables were localized to the tagged animal using angle of arrival (see details in^19^). Only clicks and buzzes assigned to the tagged whale were included in the analyses, and we followed methods in Arranz *et al*.^33^ to ensure that only foraging-related buzzes and not burst-pulses were selected. Each variable was calculated over the duration of an entire dive, with the exception of jerk peak, which was calculated over the bottom phase. Details about the derivation of these variables are published elsewhere^19,70^. These variables were selected because of their established roles in SRKW foraging activities. Slow echolocation click trains are produced during bouts of prey searching by odontocetes e.g.^29,37,39^. Buzzes occur during foraging, often just prior to prey interception^30,33,36,37^. Jerk peak, roll and heading variance have been used together to detect prey capture events with good accuracy^19^. Depth is a reliable indicator of foraging behavior, with resident-type fish-eating killer whales making deep foraging dives (i.e., often >30 m) to capture prey, particularly salmonids^35^.

### Statistical analysis

We used multivariate Hidden Markov Models (HMMs) to characterize subsurface behavior^71^. This framework has been previously established for studying behavior in cetaceans^46,47,72–75^, and is well suited for analyzing behavioral time series, as it identifies the most likely underlying, non-observable (hidden) states that produce the observed behavior. We utilized a first-order Markov process with N hidden states, in which the probability of being in the current state was determined by the previous state. The six dive variables measured during a dive defined the observed behavior for that dive. The four continuous variables (maximum depth, jerk peak, roll, heading variance) were natural log-transformed and assumed to have an approximate Gaussian distribution, and the two binary variables were modeled with a binomial distribution and logit link. All dive variables were assumed to be independent of each other, which exploratory analysis revealed was a valid assumption, and each tag deployment (tag attached to an individual) was modeled as an independent time series.

We constructed models with three to five underlying hidden states, and did not consider higher order models because the quadratic increase in model parameters would result in biologically uninterpretable models. We did not allow models with six or more states because this was not supported by field observations of SRKW behavioral activities^11,56,70,76–78^. A similar approach of constraining modeling to a pre-determined number of states has been utilized previously, to balance the tradeoffs of statistical vs. biological interpretability^47,74,75^. Models assumed that observed behavior was conditionally independent given the states (contemporaneous conditional independence). We used the depmixS4 package^79^ in R v.3.3.3 (R Core Team, Foundation for Statistical Computing, Vienna, Austria) to construct multivariate Hidden Markov Models. This platform allows for flexibility in constructing a variety of dependent mixture models. Each HMM included the six dive variables as response variables, with sex as the single covariate on each response variable. We computed the transition matrix for each model, assuming that all state transitions were possible (no elements fixed at *a priori* values), and for each model we included one of four covariates on the transition matrix: age class (juvenile or adult), sex, year or a null model without a covariate. We constructed twelve separate HMMs using unique combinations of number of states and a single transition matrix covariate; for interpretability we did not use more than one transition matrix covariate at a time.

We fit the models by maximum likelihood estimation using the expectation-maximization (EM) algorithm in the depmixS4 package, which iteratively maximizes the expected log-likelihood of model parameters given the observations and underlying states. We specified a maximum of 2000 starting values to increase the chance that a solution was found during model fitting. We fit each model 200 times from random initializations to check for numerical stability and robustness due to different starting values, and retained the model with the lowest AIC score. We compared the twelve best models via log-likelihood values and AIC scores. All 5-state models had lower AIC scores than all 4-state models, which had lower scores than all 3-state models (Table 4). The 5-state model with sex as a covariate on the transition matrix had the overall lowest AIC score, and the second lowest log-likelihood value, so we accepted this model (Table 4). Finally, we calculated transition probabilities from the state transition matrix for this model.

**Table 4:** Comparison of number of states and covariates on the transition matrix, AIC scores and log-likelihood values for all Hidden Markov models.

No. states	Transition matrix covariate	logLik	∆AIC
5	Sex	−15296.366	0
5	Age class	−15300.187	7.641
5	Year	−15285.412	18.091
5	1	−15341.371	50.011
4	Sex	−15823.431	988.131
4	Age class	−15835.986	1013.239
4	Year	−15827.778	1020.825
4	1	−15860.000	1037.268
3	Sex	−16502.042	2287.351
3	Year	−16499.977	2295.221
3	Age class	−16510.743	2304.753
3	1	−16520.492	2312.252

We analyzed results of the best HMM in R v.3.5.3. We tested whether dive duration varied by state and sex, given the increased costs of transport for females with calves^11^ and the known differences in metabolic rate and energetic requirements^12^, and the potential temporal constraints this could have on behavior. To determine whether dive duration varied by state and sex, we constructed a linear mixed model using the lme4^80^ package with dive duration (natural log-transformed to meet model assumptions) as the response variable, fixed effects of state and sex, and a random effect of deployment, followed by a Type III ANOVA test using the lmerTest package^81^ to identify significant effects, and a Tukey HSD test with adjusted p-values using the lsmeans^82^ and multcompView^83^ packages to compare levels of significant effects. To examine differences estimated between state occurrence by sex, we fit a hierarchical multinomial logistic regression model using the brms package^84^, with estimated state from the best HMM as the response variable, sex as a fixed effect, and deployment as a random effect. To determine if it was necessary to account for potential effects of deployment duration on the occurrence of the five states, we additionally fit multinomial logistic regression models as above, incorporating ln(deployment duration) as linear effects and as an offset, and compared these models to the one without deployment duration, by examining the posterior estimates of coefficients and comparing estimates using leave-one-out cross-validation (LOOIC)^85^. We found no strong support for including deployment duration as a predictor or offset in the model. Estimates of LOOIC were extremely similar across models and posterior estimates of the linear effects were uncertain, overlapping 0 in all cases, with large coefficients of variation ranging from 0.65 to 14.7. Therefore, we did not include deployment duration in the final model. The final hierarchical multinomial logistic regression model was run with four MCMC chains, a burn-in period of 2000 samples, and we retained another 1000 samples. The Rhat values of all parameters were 1.0, supporting model convergence^86^. To examine differences in cumulative time spent in each state by sex, we summed dive duration (in seconds) across state and deployment, for each sex, and implemented a Chi-square test on the cumulative time per state, between females and males.

### Ethics statement

The research was conducted in accordance with all Research Permits (USA: NMFS No. 781-1824/16163; Canada: DFO SARA/Marine Mammal License No. MML 2010-01/SARA-106B), and was approved by Northwest Fisheries Science Center’s Institutional Animal Care and Use Committee.

## Supplementary information

supplementary information

## Data Availability

The datasets analyzed during the current study are available from the corresponding author upon reasonable request.
