# Supplementary material for: Hidden Markov models reveal temporal patterns and sex differences in killer whale behavior
Source: Sci Rep. 2019 Oct 18;9:14951. doi: 10.1038/s41598-019-50942-2 (PMC6802385; doi:10.1038/s41598-019-50942-2)
Supplement: Supplementary file 1 — supplementary information [file 41598_2019_50942_MOESM1_ESM.pdf]

Supplementary Information File

Title

Hidden Markov models reveal temporal patterns and sex differences in killer whale behavior

Running title

Sex differences in killer whale behavior

Authors and Affiliations

Jennifer B. Tennessen[a, b, *], Marla M. Holt[a], Eric J. Ward[a], M. Bradley Hanson[a], Candice K. Emmons[a], Deborah A. Giles[c, 1], Jeffrey T. Hogan[d]

[a]Conservation Biology Division, Northwest Fisheries Science Center, National Marine Fisheries Service, National Oceanic and Atmospheric Administration, Seattle, WA, USA

[b] Lynker Technologies, Leesburg, VA, USA

[c]Department of Wildlife, Fish, & Conservation Biology, University of California, Davis, CA, USA,

[d]Cascadia Research Collective, Olympia, WA, USA,

[1] Present address: University of Washington, Friday Harbor Laboratories, WA, USA

*Corresponding Author:

Jennifer B. Tennessen, Northwest Fisheries Science Center, 2725 Montlake Blvd. East, Seattle, WA, 98112, USA, jennifer.tennessen@gmail.com, +1 206-860-3473

Supplementary Table S1. Estimated coefficients from a hierarchical multinomial regression model fit using the brms package in R.

	**Estimate**	**Est. Error**	**Lower 95%**	**Upper 95 %**	**Eff. Sample**	**Rhat**
mu2_intercept	2.94	0.50	1.99	4.02	3006	1
mu3_intercept	2.58	0.43	1.83	3.50	2845	1
mu4_intercept	1.61	0.70	0.13	2.83	2371	1
mu5_intercept	0.75	1.38	-2.22	3.28	2283	1
mu2_sexM	-1.59	0.70	-3.12	-0.32	2543	1
mu3_sexM	-0.68	0.56	-1.85	0.40	2554	1
mu4_sexM	0.74	0.93	-0.91	2.70	2072	1
mu5_sexM	-0.05	1.92	-3.80	3.97	2242	1

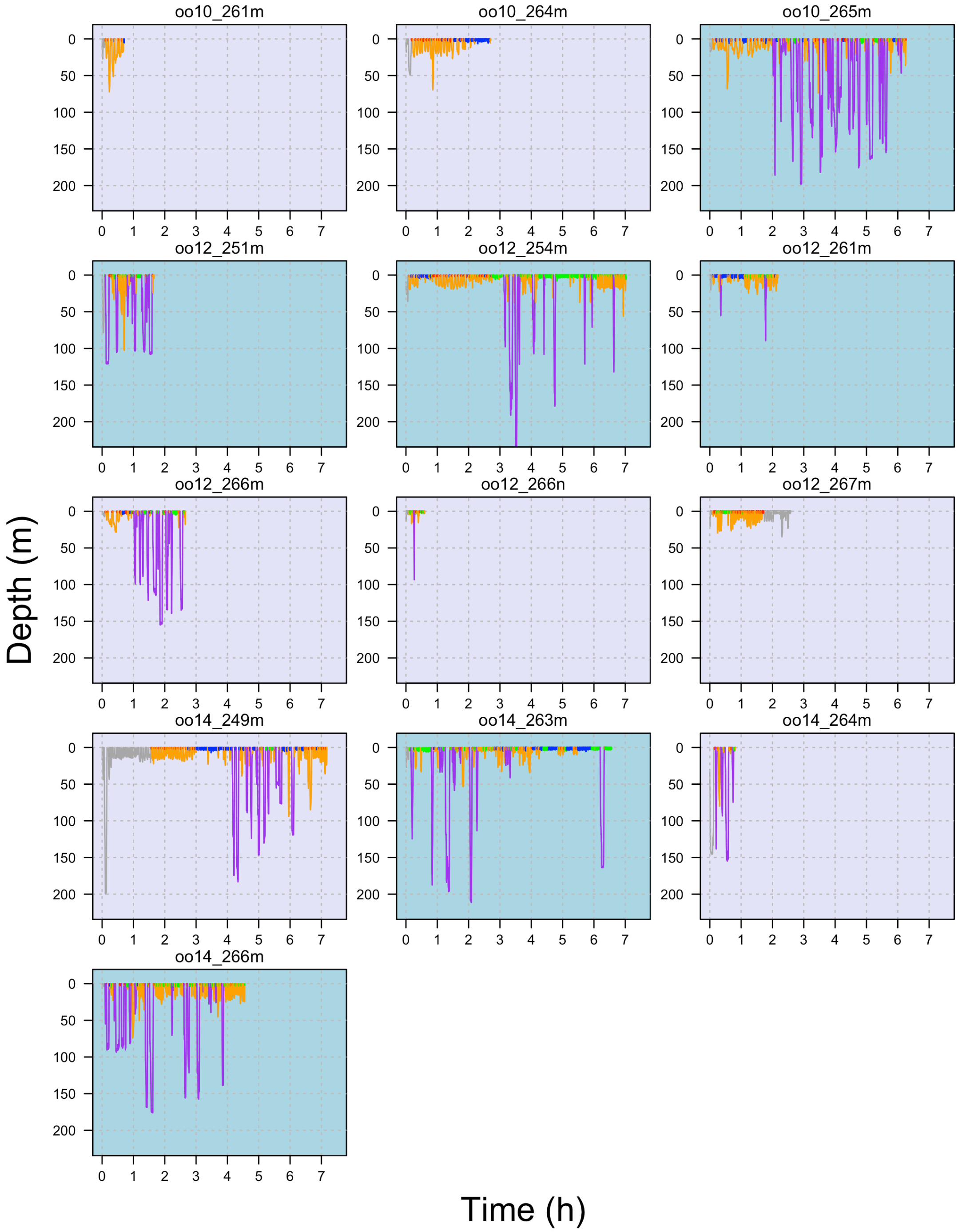

Supplementary Figure S1. Time series of behavior for each deployment indicating changes in state allocation over time. Female = lavender box, male = blue box. State 1 = purple, state 2 = red, state 3 = orange, state 4 = green, state 5 = blue. Gray dives indicate omitted 5-min interval at the start of each deployment, and periods during which acoustic data were not available.

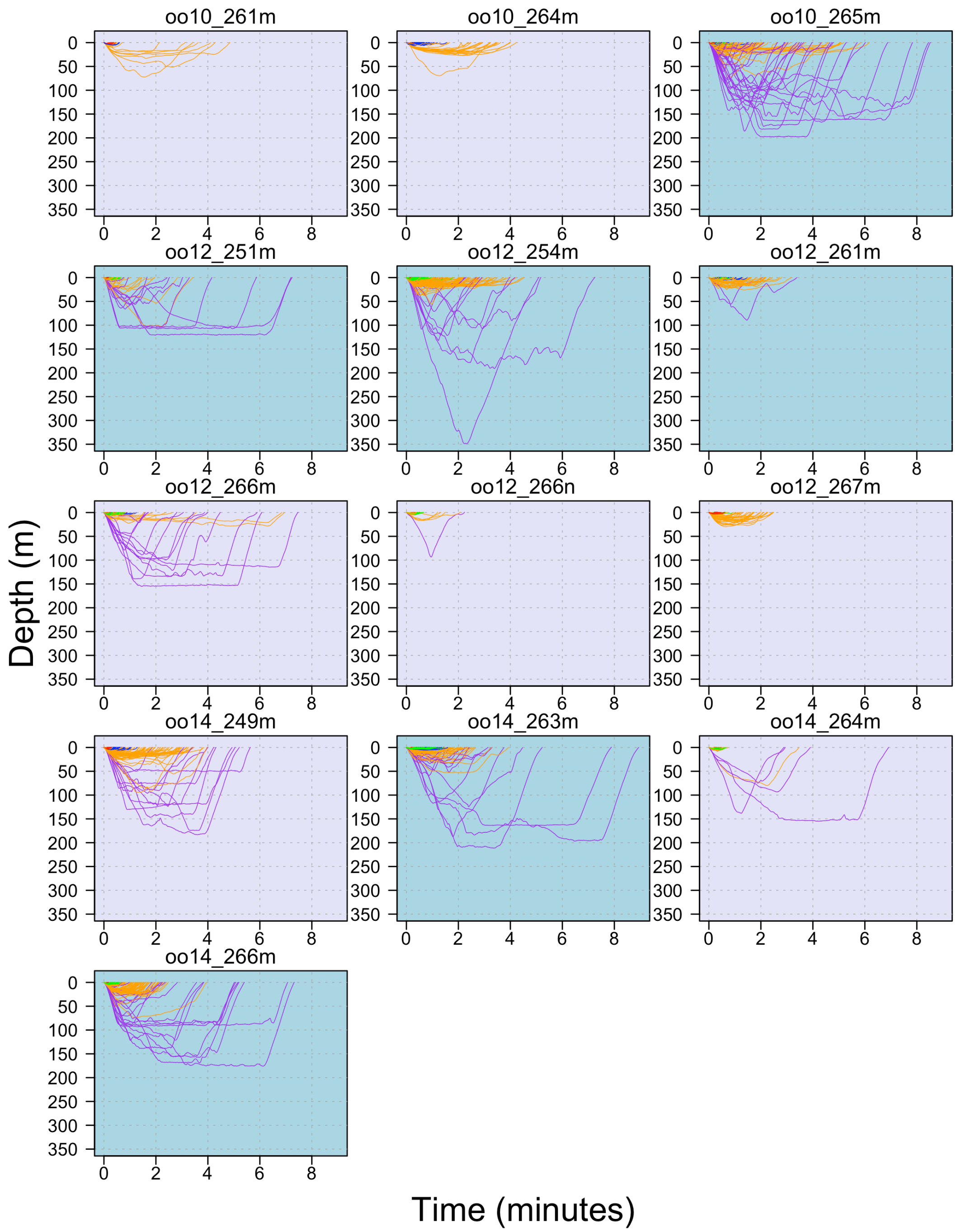

Supplementary Figure S2. State allocation, for each deployment scaled to longest dive in data set. Female = lavender box, male = blue box. State 1 = purple, state 2 = red, state 3 = orange, state 4 = green, state 5 = blue.
